# Coil Embolization Is Not Justified for Treating Patients with Veno-Occlusive Dysfunction: Case Series and Narrative Literature Review

**DOI:** 10.3390/life14070911

**Published:** 2024-07-22

**Authors:** Ko-Shih Chang, Cho-Hsing Chung, Yi-Kai Chang, Geng-Long Hsu, Mang-Hung Tsai, Jeff SC Chueh

**Affiliations:** 1 Division of Cardiovascular Medicine, Microsurgery Potency Reconstruction and Research Center, Yuan Rung Hospital, Yuanlin, Changhua 51052, Taiwan; 2 School of Nursing, National Taipei University of Nursing and Health Science, Taipei 112303, Taiwan; 3 Department of Urology, Wan Fang Hospital, Taipei Medical University, Taipei 11696, Taiwan; 4 Department of Urology, National Taiwan University Hospital, Taipei 10002, Taiwan; 5 Microsurgical Potency Reconstruction and Research Center, Hsu’s Andrology and Shu-Tien Urology Ophthalmology Clinic, Taipei 10662, Taiwan; 6 Department of Anatomy, China Medical University, Taichung 40402, Taiwan

**Keywords:** coil embolization, coil cardiac perforation, deep dorsal vein, endovascular coiling, erection-related vein, penile venous stripping, veno-occlusive dysfunction

## Abstract

**Introduction:** Herein, we explore whether coil embolization (CE) is effective in treating veno-occlusive dysfunction (VOD). We present five cases with seven CE episodes and a narrative literature review. **Methods:** From 2013 to 2018, refractory impotence prompted five men to seek penile vascular stripping (PVS), although seven CE episodes were included. All received dual cavernosography in which erection-related veins and VOD were documented. PVS entailed the venous stripping of one deep dorsal vein and two cavernosal veins. The abridged five-item version of the International Index of Erectile Function (IIEF-5) score system and the erection hardness scale (EHS) were used, and yearly postoperative follow-ups were conducted via the Internet. Using Pub Med, a narrative literature review was performed on CE treatment for VOD or varicocele. **Results:** Inserted coils were scattered along the erection-related veins, including the deep dorsal veins (n = 4), periprostatic plexus (n = 5), iliac vein (n = 5), right pulmonary artery (n = 2), left pulmonary artery (n = 2), and right ventricle (n = 1). PVS resulted in some improvements in the IIEF-5 score and EHS scale. Six articles highly recommend CE treatment for VOD. All claimed it is a minimally invasive effective treatment for varicocele. **Conclusions:** CE is not justified as a VOD treatment, regardless of its viability in the treatment of varicocele.

## 1. Introduction

Erectile dysfunction (ED) is defined as the inability to attain or maintain a rigid erection for satisfactory intercourse. It is a medical condition that affects countless males and is particularly awkward in those who are young and actively engaged in sexual activity [1,2]. Unsurprisingly, many studies of interest have been conducted and reviewed [3]. A lot of attention has been paid to married life in this context as exemplified by the studies produced during the COVID-19 pandemic [4,5]. Normal penile erection is the result of an interplay between healthy hormonal, arterial, neurological, pharmacological, systemic metabolic, psychological factors and the corpora cavernosa remaining free from cavernosal fibrosis [6]. Consequently, ED can occur if any of the said contributors is unhealthy [7].

Regarding ED treatment strategies, the discovery of phosphodiesterase type 5 (PDE-5) inhibitors, e.g., Sildenafil, opened the door for medical treatment [8], with this agent becoming the main ED treatment option Would the inventor translate the sagacious words” opportunity favors prepared mind”? [9]. However, there are many ED treatment strategies because of the paucity of ED panacea, with at least 30% of ED patients not responding to this group of agents. This number is particularly high in young males suffering from veno-occlusive dysfunction (VOD) [10]. Even though VOD is the most prevalent contributor to the ED pathophysiology, including in those whose ED results from heavy smoking [11,12], patients with VOD are typically administered oral vasodilators first and later offered second-line treatment strategies, including vacuum constriction devices, injection vasodilator therapy, endovascular therapies, or penile implantation [13]. Recently, there has been practical developments in the field of low-energy shock-wave therapy (ESWT) for ED [14], which, much like PDE-5 inhibitors before, has become extremely popular. Despite this, penile implants remain the most reliable solution among the ED treatment options listed [15], though serious complications, e.g., mechanical failure, infection, and prosthesis extrusion, still sometimes occur [16].

Penile vascular interventions aim to treat VOD or arterial insufficiency by limiting blood outflow or increasing arterial inflow in the penis [17]. Many physicians and researchers are continually seeking improvements in these procedures. To this end, pudendal stents and penile venous embolization have been introduced to treat VOD and arterial insufficiency [18]. Coil embolization (CE) has also returned as an option in the last decades. It is claimed to be an effective strategy with minimally invasive options owing to its simplicity and reproducibility [19]. Conspicuously, penile venous stripping (PVS) developed in 1986 [20], after a refined penile venous stripping treatment method won a USPTO patent on 14 August 2012 [21], many refractory young patients with VOD began requesting PVS. To fill the research gap, herein, we report five young patients who underwent penile venous CE, supplemented with a narrative literature review of CE as a treatment for VOD and varicocele.

## 2. Methods

We conducted this retrospective study with case reports and a literature review after it was approved by the institutional review board of China Medical University (CMUH112-REC1-077) on 3 May 2023. This article details five men who received CE and is supplemented by a narrative literature review. Journals in andrology, urology, radiology, and medicine-related fields were examined using MEDLINE. The search used the following terms in various combinations: erectile dysfunction, coil embolization for treating patients with erectile dysfunction, conspicuously veno-occlusive dysfunction, an older venous leak, and varicocele testis.

### Case Series of Five Patients

**Figure 1:**
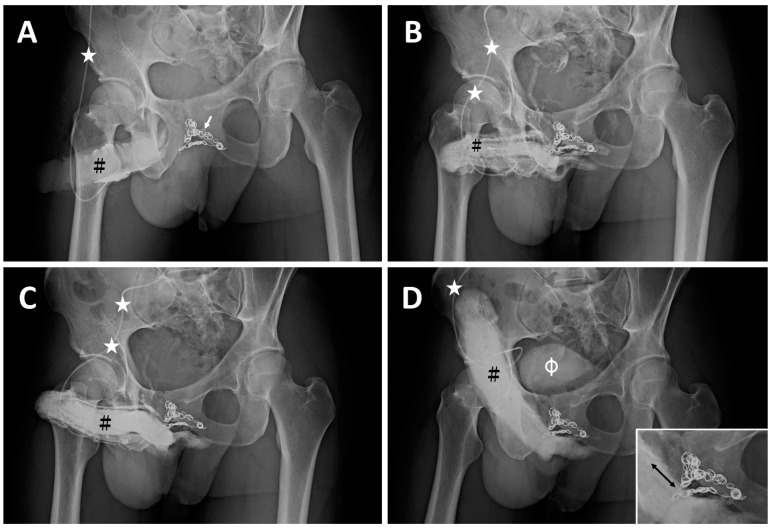
The imaging of a 33-year-old dentist. (**A**) A KUB demonstrates dozens of inserted coils (white arrow) with a 30° oblique view. The 19G scalp needle (white asterisk) was inserted into the corpora cavernosa (black hash). (**B**) After injection of 10 mL of Omipaque solution via the 19G scalp needle (white asterisk), the glans penis, corpora cavernosa, and corpus spongiosum were shown. The coils partially blocked the drainage of blood. (**C**) Further, 10 mL of solution was injected via the same scalp needle (white asterisk), and the radiopacity of the penis was enhanced. (**D**) Then, 20 mL of Omipaque solution was injected 10 min after 15 μg of prostaglandin E1 was injected intracavernously via the same scalp needle (white asterisk); slight VOD was noted; unfortunately, the erection rigidity was insufficient. Note that the proximal deep dorsal vein and cavernosal veins were engorged on the dorsal aspect of the erect penis distal to the coil (arrow). This implied the possibility of sinusoidal blood freely draining to the venous channel, resulting in engorgement and compromised rigidity. Note the urinary bladder was demonstrated (white hash).

**Figure 2:**
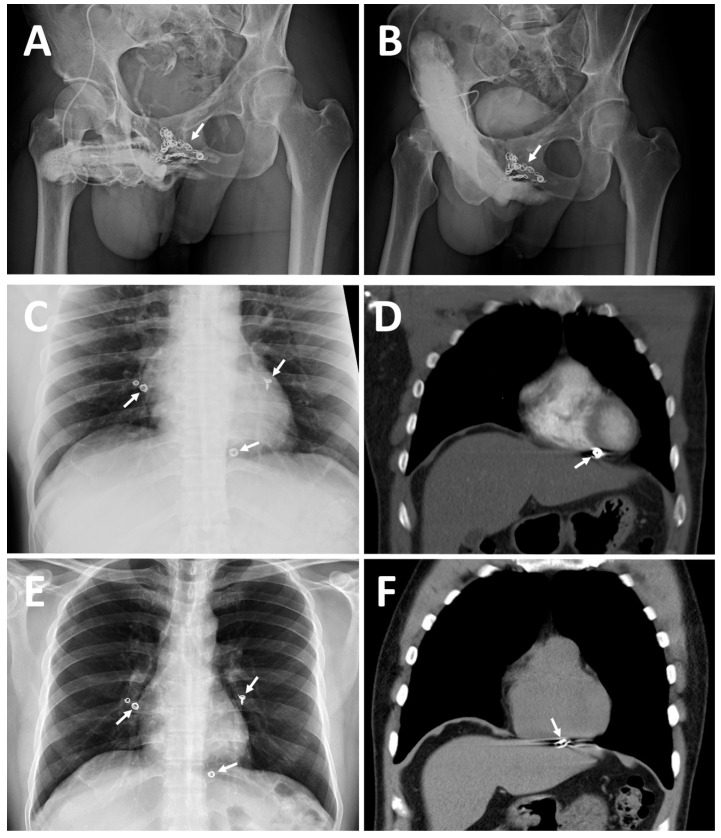
Imaging of two patients for comparison, accentuating case 2, 42-year-old housebuilder. (**A**) A 30° right oblique view KUB demonstrates dozens of inserted coils (white arrow) with excessive erection-related veins. (**B**) A total of 20 mL of Omipaque solution was injected 10 min after 15 μg prostaglandin E1 was injected intracavernously via the same scalp needle. Note that a dozen inserted coils could not specifically block the erection-related venous drainage. This was evidenced by the proximal deep dorsal vein and cavernosal veins being engorged on the dorsal aspect of the erect, but with insufficient rigidity; penis distal to the coil (white arrow). (**C**) Astonishingly, a chest X-ray film disclosed five coils scattered in the cardiothoracic cavity, with two coils lodged in the left and right pulmonary arterial trees; moreover, a coil was located in the right ventricle. (**D**) A contrast CT scan confirmed the coil stuck within the right ventricular wall (white arrow). (**E**) Thereafter, a revisit was decided upon to attempt to salvage the PVS because erectile quality improved at first after penile venous stripping. In April 2016, a follow-up chest X-ray demonstrated five lodged coils: the coils in the pulmonary artery remained stationary, and the coil in the right ventricle was continuing its migration (white arrow). (**F**) A spiral CT scan demonstrated a perforation from the right ventricle into the diaphragm, with the coil having moved through the diaphragm, before becoming stuck in the liver.

**Case 1**: A 33-year-old male dentist, born in 1979, had been suffering from very early detumescence since adolescence. He was impatient to wait for other options and resorted to penile implantation to treat his ED. Thus, he underwent penile venous coil embolization (CE) to treat veno-occlusive dysfunction (VOD) due to a long-term venous leak in 2011. Unfortunately, after the coil implant, the early detumescence remained the same and poorer erection rigidity ensued; he was referred for our medical assistance in 2012. A dual pharmaco-cavernosography scout film demonstrated several dozen coils (Figure 1A) lodged within the periprostatic venous plexus (Figure 1B), and VOD was confirmed in a corresponding pharmaco-cavernosography (Figure 1C,D). The coils remained in situ at the patient’s last evaluation (Figure 2A,B).

**Case 2**: A 42-year-old housebuilder, born in 1972, had suffered from ED resulting from VOD since 1994 and received penile venous CE in 2000. He experienced some improvement in erection quality from 2000 to 2003, but then ED recurred, with a subsequent physician declining his request for CE in 2005. Refractory ED prompted him to seek out medical assistance for which he traveled over 10,000 km in November 2014. Pharmarco-cavernosography demonstrated significant VOD. Surprisingly, although a pelvic X-ray failed to find coil imaging, a chest X-ray disclosed five coils lodged in pulmonary arteries (Figure 2B).

A contrast CT scan confirmed two coils installed in the left and right pulmonary arterial trees. The coils were tightly adhesive to the vessel wall; a coil had traversed the right ventricle wall and lodged within the pericardium (Figure 2C). He received PVS of the excessive drainage veins with an uneventful postoperative course. He experienced a significant improvement parallel to a postoperative cavernosogram with enhanced intracorporeal retention, although it was not entirely satisfactory. Then, a revisit was decided upon to salvage the PVS in April 2016. A chest X-ray (Figure 2E) and spiral CT demonstrated further migration and perforation of the right ventricle wall and diaphragm by the coil (Figure 2F). The liver border was irregular and penetrated, and cirrhosis was incidentally noted. He eventually benefited somewhat from our ambulatory surgery, although our primary concern was morbidity from the uncontrolled coil. 

**Figure 3:**
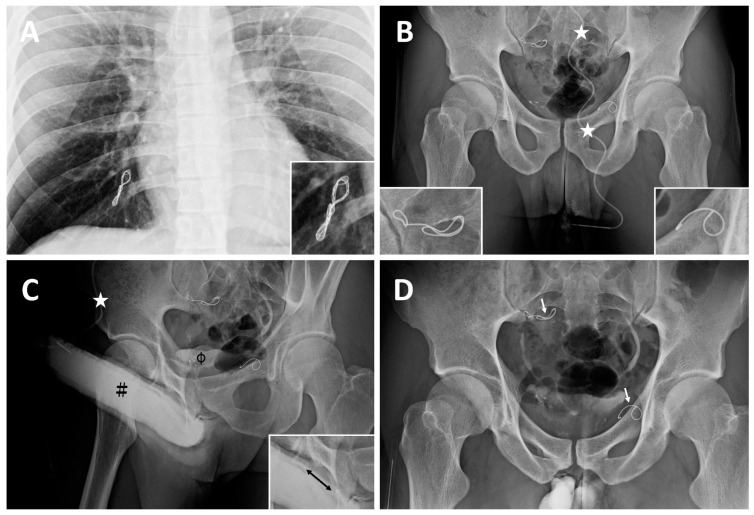
Imaging of a banker born in 1985. (**A**) In August 2016, a chest X-ray disclosed a dumbbell-shaped twisted coil complex lodged in the right pulmonary arterial avenues (magnified insert; bottom right). It was inserted in 2008 for the first CE. (**B**) A 19G scalp needle (white asterisk) was fixed while the needle tip was inserted intracavrnously. A KUB showed two coils lodged at the bifurcation level of the right iliac vein (magnified in the insert; bottom right) and left internal pudendal vein (magnified in the insert; bottom left), which corresponded to the coils inserted in 2010 and 2012, respectively. Those two coils were noted to be outside the venous channels as they were not in the venous vessel course but perpendicular to it. (**C**) Although artificial erection was induced via PGE-1 vasoactive agent, its rigidity was undermined by erection-related veins (black double-head arrow; bottom left insert) mounting the dorsal groove concave of the corpora cavernosa (black pump) and urinary tract (black hash). (**D**) After penile venous stripping, intracorporeal retention was pronounced at the penile crura and the coils remained stationary. Note a tapeworm-shaped coil was noted in the region of the right iliac vein (white arrow) and a pigtail-shaped coil was shown at the left internal pudendal vein level (white arrow), they are at 90 degrees to the venous channel.

**Case 3**: A 31-year-old banker, born in 1985, had suffered from ED resulting from VOD since age 23. He then underwent his first penile venous CE in the northern hemisphere in 2010, with no improvement in erectile function. Table 1 summarizes three CE courses at different medical school-affiliated hospitals. Acute chest pain led him to the emergency room. From an imaging study, he was told that a dumbbell-shaped coil complex (Figure 3A) was evident in the right pulmonary artery eight days postoperatively. Refractory ED prompted him to receive second and third penile venous CE procedures in 2012 and 2014 at different medical schools. Tapeworm-shaped and pigtail-shaped coils were demonstrated (Figure 3B). His ED did not improve. Refractory ED prompted him to seek out medical assistance after a long trip in August 2016. A chest X-ray confirmed a twisted coil complex lodged in the right pulmonary artery, although this patient had experienced no more chest pain since the painful episode in 2010. An abdominal x-ray showed two coils at 90-degree to the body axis: one lodged in the bifurcation region of the left iliac region and the other installed in the right internal pudendal vein level (Figure 3B), which corresponded to the coils inserted in 2012 and 2014, respectively. Dual pharmaco-cavernosography showed that the coils did not fit within the internal iliac or pudendal veins regardless of VOD (Figure 3C). He had an uneventful course after PVS, which caused significant intracorporeal retention, conspicuously in the penile crura (Figure 3D), and flew home on the third postoperative day. Erectile functional improvement is ongoing, and he has resumed sexual activity.

**Figure 4:**
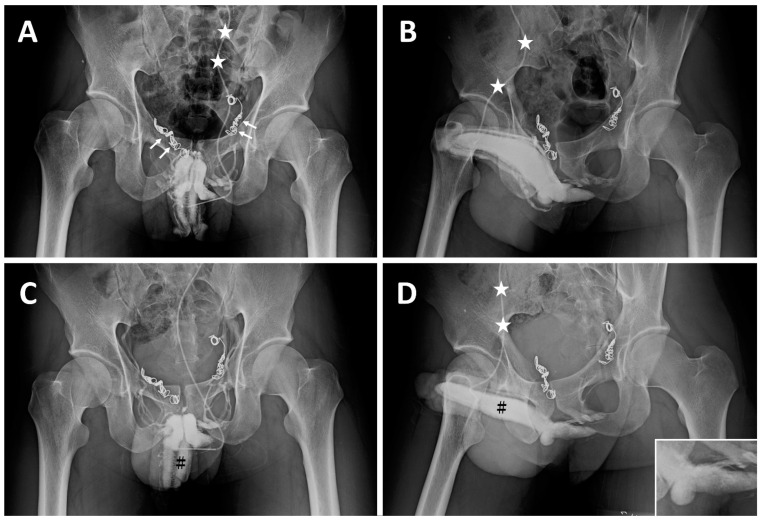
Imaging of a sophomore student born in 1993. (**A**) AP view of a preoperative cavernosogram demonstrated coil plexus inserted in the right periprostatic plexus (two white arrows) and left internal pudendal veins (two white arrows) with incomplete blockage of the venous channel. The 19G scalp needle was fixed (white asterisk) ready for injection. (**B**) Injection of Omipaque via the same scalp needle (white asterisk), a late cavernosogram of a 30° oblique view is shown. Again, the venous blockage was partial. (**C**) After all emissary’s veins were rigidly fixed at the tunic level, penile venous stripping was conducted, and a cavernosogram demonstrated the corpora cavernosa (black hash) exclusively; note the venous channels are present in A but absent in this film. (**D**) Again, those drainage veins were blocked by surgery. Consequently, intracorporeal retention was readily confirmed as the injected opaque region was confined to the corpora cavernosa (black hash). A comparison was made with (**B**), whereby the drainage veins were stripped off.

**Case 4 and 5:** Two university students, born in 1992 and 1993, respectively, had suffered from ED resulting from VOD since the ages of 23 and 21. They underwent their first penile venous CE in the northern hemisphere in 2015 and 2017 with no improvement in erectile function. In 2017 and 2018, intractable ED prompted them to visit our institute, where many inserted coils were noted in the proximal deep dorsal vein (Figure 4A) and the superficial dorsal vein (Figure 4B). Then, they had an uneventful course after PVS (Figure 4C,D) and flew home on the third and fourth postoperative days. Erectile functional improvement was noted, and sexual activity was resumed.

## 3. Results

Inserted coils were seen inside and outside the cardiovascular circulation system, particularly in the erection-related veins of the de novo penile fibro-vascular assembly to the pulmonary arteries, including the deep dorsal veins (n = 4), periprostatic plexus (n = 5), iliac vein (n = 5), right pulmonary artery (n = 2), left pulmonary artery (n = 2), and right ventricle (n = 1), with cardiac wall perforation being noted within one and half years. The average follow-up period was 6.3 ± 2.1 years. Even though the radiopacity was marginally enhanced after penile venous stripping, certain objective improvements were evidenced in the imaging data and the IIEF-5 score and EHS scale. Regarding publications on coil therapy for treating VOD, six articles recommend it highly, whereas one regards it as an acceptable option. All claim it as minimally invasive and effective. In addition, EC for the treatment of varicocele testis is consistently recommended parallel to brain vasculature treatment.

### Literature Review of Endovascular Coil Embolization

Vascular diseases occur throughout the human body because every organ system requires the nutrition that a functional cardiovascular system supplies [22]. As a rule, the amount of drainage blood should equal the amount supplied to each independent organ. Given that no one can avoid vascular disorders, most men will confront the ED issue in their lives. Like other organs, in the human penis, vascular disorders can be categorized into arterial, venous insufficiency, and a mixed-type disorder. In recent decades, every professional has benefited from advanced technologies and research themes as was exemplified by the COVID-19 vaccine [23]. Similarly, state-of-the-art tools have been widely developed to reduce invasiveness in every subspecialist surgery and the related procedures are, thus, collectively categorized as minimally invasive techniques [24]. Endovascular intervention is one typical example. An embolus could be of natural origin, such as a pathological bolus in the pulmonary embolism, or an artificial device for therapeutic purposes, such as in the hemostatic treatment of idiopathic intractable epistaxis [25]. Another practice is that of blocking a tumor-feeding artery to starve the tumor cells [26]. Overall, surgery has a more versatile role in clinical medicine than ever before and has become the mainstay of interventional radiology practice because of its simplicity and reproducibility [27].

Despite endovascular coiling being developed with multidisciplinary collaboration through the synthesis of several innovations between 1970 and 1990 in the field of electronics, neurosurgery, and interventional radiology [28], it does not appear to be applicable for ED resulting from penile arterial insufficiency that would not benefit from angioplasty or an arterial stent. In addition, we assessed the literature on CE for treating ED resulting from VOD, which is commonly believed to be a minimally invasive effective strategy. Furthermore, we analyzed various representative articles covering coiled embolization for varicocele testis [29], which is so common that it is used in commonplace issues such as hemorrhoids [30]. To build a comprehensive overview, Table 2 provides a general summary. With a sample size of just 18, Courtheoux published an article on coiled embolization for treating ED resulting from VOD in which embolization treatment success was reported in 15 of 17 (83.3%) patients in 1985 [31]. He later reported that 26 patients benefited from this procedure from a group of 40, with a 65% success rate. He concluded that this technique was simple, safe, and effective in treating impotence caused by venous leakage in 1986 [32]. A similar article was published in a different language in 1987 [33]. In 1991, the sample size was expanded to 46 patients, with a success rate of 57% after eight-month follow-up [34]. However, in 1994, Schild reported a success rate of just 22.2% among 19 patients with a 25-month follow-up [35]. In 1998, Malossini used coil embolization plus partial resection of the proximal venous trunk on 17 patients and noted 11 patients with a successful outcome from 19 males being followed-up [36]. In 2014, Rebonato performed sclerosing embolization on patients who underwent coiled embolization, reporting that 7 males out of 18 benefited in a follow-up period of 13.3 months [37]. Carrino applied sclerosing embolization with the Valsalva maneuver on 171 patients with a 77.2% success rate, with a follow-up for six months [38]; however, only the abstract is available. The full article would be of considerable interest since the case number is as large as 171. Regarding varicocele, coil embolization appears to be more effective in many publications; here, we analyze a representative article for comparison. In 2009, Bechara reported a success rate of 95% on 41 patients in a follow-up period of 39.3 months [39]. Moreover, Kutlu reported the application of coil embolization in 32 patients with varicocele, although migration occurred [40]. In 2018, Markris published a review article on coil embolization in 898 patients with varicocele with a 90.9% success rate [41].

In summary, advanced medicine takes advantage of material science and engineering, leading to advances in clinical applications, as will be the case with artificial intelligence. In this manner, physicians can develop more accessible and effective strategies for confronting human diseases. Therefore, coil embolization deserves to be explored, especially as varicocele and VOD are the most common disease entities in urology. In the medical literature, six articles highly recommend CE for treating VOD and long-term venous leak, claiming to be minimally invasive and effective, but none of these are from the recent decades. In contrast, CE is recommended for treating varicocele, although coil migration to the right heart ventricle can occur.

## 4. Discussion

Endovascular coiling can be successfully applied to reduce blood circulation to brain aneurysms using microsurgical detachable platinum wires, with the clinician inserting one or more into the aneurysm until it is determined that blood flow is no longer occurring within the space. While the procedure itself has been and continues to be compared to surgical clipping, the development of the concept and procedure has resulted in it becoming a practice in many centers and subspecialist fields [42]. In the human body, many veins, especially those in the legs, penis, and testicular veins, have one-way valves. Each valve consists of two cusps with edges that meet. When blood flow moves toward the heart, it pushes the cusps open like one-way swinging doors. If gravity or muscle contractions try to pull the blood backward or if blood begins to back up in a vein, the cusps are pushed closed, preventing backward flow [43]. As the venous wall can only sustain a low pressure, it is understandable penile erection rigidity (Figure 1) can be compromised because all emissary veins communicate freely between the cavernosal sinusoids and periprostatic venous plexus. Thus, the endovascular coiling procedure can establish a environment whereby pressure–vein communication is unrestricted in a corpora cavernosa suffering from VOD. The question is, therefore, how is erection rigidity attainable? In our clinical experience and after assessing six cases of endovascular sclerotherapy, the venous occlusion location is represented by the emissary veins close to the outer tunica, which is an exclusive physiological way to establish the corpora cavernosa (CC) in order to free them from VOD. In addition, the surgical method should aim to cure VOD rather than partially blocking vessels far from the CC chamber as a result of CE. Thus, endovascular interventions for treating VOD warrants further scientific study in the future.

In the literature review, endovascular coiling was shown to be appropriate for treating varicocele testis because the blood flow does not vary much based on physiological requirements. Astonishingly, a migration of the coil in the right ventricle was reported [44]. Despite the large patient population having undergone endovascular coiling for varicocele testis and the rate of coil migration being so rare, this extreme complication does occur and deserves to be monitored. In contrast, despite the small patient population having undergone endovascular coiling for VOD issues, uncontrolled coil migration to the pulmonary artery was reported as early as 1993 [45]. We were consulted by six patients who underwent endovascular coiling to treat VOD from 2012 to 2017, with five of them visiting our institute in person six times. In these patients, not only did endovascular coiling have little benefit for their ED issues, but extraordinary complications also occurred, such as coil migration along the pulmonary artery tree and into the lower right pulmonary artery within one week, coil migration into the right ventricle within five years, and further cardiac wall penetration in one and half years thereafter (Figure 2). We believe that potential patients should be informed about these risks as the heart and cardiovascular system are involved. Although we are unaware of a method of avoiding coil migration, we feel the sharpness of the coil edge may have contributed to its migration out of the vessel in the case of the 31-year-old banker.

Endovascular coiling may be an easy treatment option for physicians confronted with a patient with VOD owing to its simplicity, reproducibility, and low morbidity. Moreover, it is effective and safe in various vascular diseases and would be a positive option if functional for VOD treatment. Among venous treatment options, penile venous embolization is an old method that is now being newly assessed from the perspective of interventional radiology. It appears safe, although the surprising morbidity of the 42-year-old house builder and 31-year-old banker described herein underscores the need for caution [46]. Human erectile rigidity only occurs if the corpora cavernosa is healthy (CC). The CC sinusoids are encircled by the bi-layered tunica albuginea, which is essential in the erection mechanism. On full erection, CC arterial inflow increases from 2–3 mL/min to 60–80 mL/min, which is the same in the CC suffering from VOD. This implies that a penile venous embolization coil is at risk of migrating throughout the body once it is inserted in the main common drainage channel of the CC.

Recent research described the penile fibro-vascular assembly as a new frontier of the human cardiovascular system [47]. The penile venous anatomy includes one deep dorsal vein, two cavernosal veins, and two pairs of para-arterial veins (as opposed to one single vein), all situated between Buck’s fascia and tunica. These erection-related veins are the principal components in erectile rigidity and drain the sinusoidal blood out of the corpora cavernosa, before becoming confluent to the periprostatic venous plexus. Moreover, large patient populations seek and benefit from anatomy-based penile venous stripping surgery for ED resulting from veno-occlusive dysfunction. The ideal solution for treating penile veno-occlusive dysfunction should limit blood flow close to the tunic outer longitudinal layer, where the veno-occlusive mechanism operates [48]. This implies that an effective strategy for treating penile erection-related veins should aim at the level closest to the more extended longitudinal tunica albuginea layer. Accordingly, the efficacy of venous embolization warrants further research because it only treats the vascular channels outside the corpora cavernosa, which may be why penile venous embolization is ineffective, as evidenced in this five-patient case series.

## 5. Conclusions

In summary, we found that migrating coils can flow through the cardiovascular systemic circulation until they encounter an impedance. Thus, penile endovascular coiling may incur the risk of extreme complications, including cardiac wall penetration, and patients should be informed of this possibility before choosing coil embolization to treat ED resulting from VOD. Our study has certain limitations, including the small sample size (five patients) and its retrospective nature.

## Figures and Tables

**Table 1:** A man, born in 1985, sustained coils migrating from his veins and arriving in the right pulmonary artery at three different medical universities.

Item	Occurred Time	Symptoms	Signs
ED ^a^	2008	Early detumescence of rigid erection, position-dependent	A multiplanar diagnosis workup showed a veno-occlusive dysfunction.
CE(I) ^b^	2010	Same	Acute chest pain was caused by the migration of a dumbbell-shaped coil to the right pulmonary artery.
CE(II) ^c^	November 2012	Same	A tapeworm-shaped coil was noted in the region of the right iliac vein at 90 degrees to the venous channel.
CE(III) ^d^	May 2014	Same	A pigtail-shaped coil was shown at the left internal pudendal vein level at 90 degrees to the venous channel.

^a^ ED—erectile dysfunction; CE—coil embolization; (I) ^b^, (II) ^c^, and (III) ^d^ denote the first, second, and third episodes of receiving CE, respectively.

**Table 2:** Endovascular embolization in treating patients with venous leak or varicocele.

Author/Publication (Reference Number)	Patient No. VL/Varicocele	Therapy Method	Follow-Up (Months)	Success Rate %/Follow Available
Courtheoux/1985 [31]	17	VL ^a^ (CE) ^b^	?	83.3 (15/18)
Courtheoux/1986 [32]	40	VL(CE) ^b^	?	65.0 (26)
Courtheoux/1991 [34]	46	VL(CE) ^b^	8	57
Schild/1994 [35]	19	VL(CE) ^b^	25	22.2 (24)
Malossini/1998 [36]	17	VL CR ^c^	NA	73.4 (11/15)
Bechara/2009 [39]	41	Varicocele (CE)	39.3	95
Kutlu/2009 [19]	32	Varicocele (CE)	?	Migration
Makris/2018 [41]	898	Varicocele	?	90.9 (9.1)
Total	139/971	?	?	?

**^a^** VL—venous leakage. **^b^** CE—coil embolization. **^c^** CR—coiling and resecting some veins simultaneously. ?: not mentioned in the reference.

## Data Availability

The data for this research article were taken from the decades-old repository of clinical records and anonymized.

## References

[1] Burnett A.L., Nehra A., Breau R.H. Erectile Dysfunction: AUA Guideline (2018). American Urological Association Website. https://www.auanet.org/guidelines/erectile-dysfunction-aua-guideline-(2018).

[2] Calzo J.P., Austin S.B., Charlton B.M., Missmer S.A., Kathrins M., Gaskins A.J., Chavarro J.E. (2021). Erectile Dysfunction in a Sample of Sexually Active Young Adult Men from a U.S. Cohort: Demographic, Metabolic and Mental Health Correlates. J. Urol..

[3] Kessler A., Sollie S., Challacombe B., Briggs K., Van Hemelrijck M. (2019). The global prevalence of erectile dysfunction: A review. BJU Int..

[4] Fang D., Peng J., Liao S., Tang Y., Cui W., Yuan Y., Wu D., Hu B., Wang R., Song W. (2021). An Online Questionnaire Survey on the Sexual Life and Sexual Function of Chinese Adult Men During the Coronavirus Disease 2019 Epidemic. Sex. Med..

[5] Sansone A., Mollaioli D., Ciocca G., Colonnello E., Limoncin E., Balercia G., Jannini E.A. (2021). “Mask up to keep it up”: Preliminary evidence of the association between erectile dysfunction and COVID-19. Andrology.

[6] Andersson K.E., Wagner G. (1995). Physiology of penile erection. Physiol. Rev..

[7] Kaminetsky J. (2008). Epidemiology and pathophysiology of male sexual dysfunction. Int. J. Impot. Res..

[8] (1998). FDA approves oral therapy for erectile dysfunction. Am. J. Health Syst. Pharm..

[9] (1854). Dans les champs de l’observation le hasard ne favorise que les esprits préparés. Lecture: *University of Lille*.

[10] Papagiannopoulos D., Khare N., Nehra A. (2015). Evaluation of young men with organic erectile dysfunction. Asian J. Androl..

[11] Fuchs A.M., Mehringer C.M., Rajfer J. (1989). Anatomy of penile venous drainage in potent and impotent men during cavernosography. J. Urol..

[12] Elhanbly S., Abdel-Gaber S., Fathy H., El-Bayoumi Y., Wald M., Niederberger C.S. (2004). Erectile dysfunction in smoker. A penile dynamic and vascular study. J. Androl..

[13] Hatzimouratidis K., Giuliano F., Moncada I., Muneer A., Salonia A., Verze P. (2018). EAU Guidelines on Erectile Dysfunction, Premature Ejaculation, Penile Curvature and Priapism. https://d56bochluxqnz.cloudfront.net/media/16-Male-Sexual-Dysfunction_2017_web.pdf.

[14] Vardi Y., Appel B., Kilchevsky A., Gruenwald I. (2012). Does low intensity extracorporeal shock wave therapy have a physiological effect on erectile function? Short-term results of a randomized, double-blind, sham controlled study. J. Urol..

[15] Frydman V., Pinar U., Abdessater M., Akakpo W., Grande P., Audouin M., Mozer P., Chartier-Kastler E., Seisen T., Roupret M. (2021). Long-term outcomes after penile prosthesis placement for the Management of Erectile Dysfunction: A single-Centre experience. Basic Clin. Androl..

[16] Bettocchi C., Ditonno P., Palumbo F., Lucarelli G., Garaffa G., Giammusso B., Battaglia M. (2008). Penile Prosthesis: What Should We Do about Complications?. Adv. Urol..

[17] Shishehbor M.H., Philip F. (2012). Endovascular treatment for erectile dysfunction: An old paradigm revisited. J. Am. Coll. Cardiol..

[18] Kutlu R., Soylu A. (2009). Deep dorsal vein embolization with N-butyl-2-cyanoacrylate and lipiodol mixture in venogenic erectile dysfunction: Early and late result. Radiol. Oncol..

[19] Kim E.D., Owen R.C., White G.S., Elkelany O.O., Rahnema C.D. (2015). Endovascular treatment of vasculogenic erectile dysfunction. Asian J. Androl..

[20] Huang P.-C., Hsu G.-L., Skinner M.K. (2018). Vascular Surgery for Erectile Dysfunction. Encyclopedia of Reproduction.

[21] Hsu G.L. (2012). Physiological Approach to Penile Venous Stripping Surgical Procedure for Patients with Erectile Dysfunction. U.S. Patent.

[22] Schultz S.G. (2002). William Harvey and the Circulation of the Blood: The Birth of a Scientific Revolution and Modern Physiology. N. Physiol. Sci..

[23] Pardi N., Hogan M.J., Porter F.W., Weissman D. (2018). mRNA vaccines—A new era in vaccinology. Nat. Rev. Drug Discov..

[24] Wickham J.E.A., Fitzpatrick J.M. (1990). Minimally Invasive Surgery. Br. J. Surg..

[25] Vitek J. (1991). Idiopathic intractable epistaxis: Endovascular therapy. Radiology.

[26] White R.I. (1984). Embolotherapy in vascular disease. AJR Am. J. Roentgenol..

[27] Coldwell D.M., Stokes K.R., Yakes W.F. (1994). Embolotherapy: Agents, clinical applications, and techniques. Radiographics.

[28] Guglielmi G. (2009). History of the genesis of detachable coils. J. Neurosurg..

[29] Kroese A.C., de Lange N.M., Collins J.A., Evers J.L. (2013). Varicocele surgery, new evidence. Hum. Reprod. Update.

[30] Kaidar-Person O., Person B., Wexner S.D. (2007). Hemorrhoidal disease: A comprehensive review. J. Am. Coll. Surg..

[31] Courtheoux P., Maiza D., Henriet J.P., Alachkar F., Solassol A., Maiza C., Théron J. (1985). Correction des insuffisances érectiles d’origine veineuse par ballonnets largables et coils. J. Radiol..

[32] Courtheoux P., Maiza D., Henriet J.P., Vaislic C.D., Evrard C., Theron J. (1986). Erectile dysfunction caused by venous leakage: Treatment with detachable balloons and coils. Radiology.

[33] Courtheoux P., Maiza D., Henriet J.P., Maiza C., Mani J., Pelouze G., Theron J. (1987). Exploration et traitement par voie endovasculaire des insuffisances érectiles d’origine veineuse [Study and treatment using an endovascular approach of erectile insufficiency of venous origin]. J. Urol..

[34] Courtheoux P. (1991). Embolisation des insuffisances érectiles par fuite veineuse [Embolization in venous erectile insufficiency]. Ann. Urol..

[35] Schild H.H., Mildenberger P., Kersjes W. (1994). Effectiveness of platinum wire microcoils for venous occlusion: A study on patients treated for venogenic impotence. Cardiovasc. Intervent. Radiol..

[36] Malossini G., Ficarra V., Cavalleri S., Morana G., Zanon G., Mansueto G.C. (1998). Risultati a lungo termine del trattamento percutaneo veno-occlusivo della disfunzione erettile di origine venosa [Long-term results of the veno-occlusive percutaneous treatment of erectile disorders of venous origin]. Arch. Ital. Urol. Androl..

[37] Rebonato A., Auci A., Sanguinetti F., Maiettini D., Rossi M., Brunese L., Carrafiello G., Torri T. (2014). Embolization of the periprostatic venous plexus for erectile dysfunction resulting from venous leakage. J. Vasc. Interv. Radiol..

[38] Carrino M., Pucci P., Chiancone F., Battaglia G., Meccariello C. (2016). Embolization of the deep dorsal vein using aethoxysklerol in the treatment of erectile dysfunction resulting from venous leakage. Analysis of our experience with 171 patients. XXIII Congresso Nationale Auro.it.

[39] Bechara C.F., Weakley S.M., Kougias P., Athamneh H., Duffy P., Khera M., Kobayashi K., Lin P.H. (2009). Percutaneous treatment of varicocele with micro-coil embolization: Comparison of treatment outcome with laparoscopic varicocelectomy. Vascular.

[40] Ficarra V., Novara G., Artibani W., Cestari A., Galfano A., Graefen M., Guazzoni G., Guillonneau B., Menon M., Montorsi F. (2009). Retropubic, laparoscopic, and robot-assisted radical prostatectomy: A systematic review and cumulative analysis of comparative studies. Eur. Urol..

[41] Makris G.C., Efthymiou E., Little M., Boardman P., Anthony S., Uberoi R., Tapping C. (2018). Safety and effectiveness of the different types of embolic materials for the treatment of testicular varicoceles: A systematic review. Br. J. Radiol..

[42] Babiker M.H., Gonzalez L.F., Albuquerque F., Collins D., Elvikis A., Zwart C., Roszelle B., Frakes D.H. (2013). An In Vitro Study of Pulsatile Fluid Dynamics in Intracranial Aneurysm Models Treated with Embolic Coils and Flow Diverters. IEEE Trans. Biomed. Eng..

[43] Helps E.P., McDonald D.A. (1954). Observations on laminar flow in veins. J. Physiol..

[44] Karia N., Balmforth D., Lall K., Gupta S., Bhattacharyya S. (2020). Migration of a Varicocele Coil to the Right Heart. JACC.

[45] Moriel E.Z., Mehringer C.M., Schwartz M., Rajfer J. (1993). Pulmonary migration of coils inserted for treatment of erectile dysfunction caused by venous leakage. J. Urol..

[46] Hsu G.L., Chang Y.K., Chiang I.N., Hsu C.-Y., Chang H.-C., Chueh S.C.J. (2022). A case report of right cardiac ventricle perforation by uncontrolled embolization coil inserted for treating penile veno-occlusive dysfunction. Urol. Case Rep..

[47] Hsu G.L., Chang H.C., Molodysky E., Hsu C.Y., Tsai M.H., Yin J.H., Chen M.T. The penile fibro-vascular assembly is the last remaining independent vascular compartment to be elucidated in the entire human body. Proceedings of the 22nd International Society of Sexual Medicine World Webinar Meeting.

[48] Hsu G.-L., Lu H.-C., Skinner M.K. (2018). Penis Structure—Erection. Encyclopedia of Reproduction.

